# Zinc sulphate attenuates metabolic dysfunctions induced by olanzapine via the reduction of insulin resistance, hepatic oxidative stress, and inflammation in albino rats

**DOI:** 10.1186/s40360-025-00889-0

**Published:** 2025-04-14

**Authors:** Samir A. E. Bashandy, Rasha E. Mostafa, Marawan A. El-Baset, Fatma A. A. Ibrahim, Fatma A. Morsy, Omar A. Farid, Halima M. Ibrahim, Bassim M. S. A. Mohamed

**Affiliations:** 1 Pharmacology Department, Medical Research and Clinical Studies Institute, National Research Centre, 33 El-Bohouth St., Dokki, P.O. 12622, Cairo, Egypt; 2 Biochemistry Department, Biotechnology Research Institute, National Research Centre, 33 El- Bohouth St., Dokki, P.O. 12622, Cairo, Egypt; 3 Pathology Department, Medical Research and Clinical Studies Institute, National Research Centre, 33 El-Bohouth St., Dokki, P.O. 12622, Cairo, Egypt; 4 Physiology Department, National Organization for Drug Control and Research, Cairo, Egypt; 5 Department of Neurology and Stark Neurosciences Research Institute, Indiana University School of Medicine, Indianapolis, IN USA

**Keywords:** Zinc sulphate, Olanzapine, Metabolic dysfunction, Hepatic oxidative stress, Insulin resistance, Inflammation

## Abstract

Olanzapine, an atypical antipsychotic drug, is used to treat psychological diseases. However, it’s use carries common side effects. Those include weight gain, dyslipidemia, elevated glucose levels, and disrupted oxidative balance. We aimed to test the effect of zinc coadministration to lessen metabolic disturbances, inflammation and oxidative stress in a rat model. Four treatment groups (*n* = 6) were involved in this investigation. Group 1 was the control group (received no intervention). Group 2 received olanzapine (10 mg/kg, p.o.; daily) for six weeks, whereas Groups 3 and 4 received 50 mg/kg and 100 mg/kg of zinc sulphate (ZnSO_4_,p.o.; daily) respectively, in addition to olanzapine (10 mg/kg p.o.; daily). Following treatment completion, group 2 showed increased levels of stress markers (GSSG, MDA, and NO) and impaired levels of antioxidant markers (CAT, SOD, and GSH). Further, a strong positive correlation between insulin resistance index (HOMA-IR) and IL-6, TNF-α, and MDA of liver. Insulin resistance is a possible manifestation of the oxidative stress burden and the widespread inflammatory environment. In groups 3 and 4, however, ZnSO_4_ recovered each of these markers in a dose-dependent manner. Improvements were also noted in other homeostatic markers, such as taurine, coenzyme Q10, ascorbic acid, and vitamin E. Remarkably, in both combination groups, there was a significant improvement in all metabolic indicators of dyslipidemia (triglycerides, total cholesterol) and insulin resistance index. The biochemical study and the histological assessment of the liver slices agreed with the results. Thus, the results clearly suggest that Zinc supplementation can significantly improve oxidative stress, inflammation, metabolic perturbation (dyslipidemia and insulin resistance), and liver injury caused by olanzapine in Albino rats.

## Introduction

Olanzapine, classified as atypical antipsychotic pharmaceutical agent, is administered for the management of psychological disorders [1]. However, olanzapine may be associated with oxidative tissue damage and alteration of trace element metabolism [2], thereby resulting in a myriad of complex health complications. Moreover, the administration of atypical antipsychotics has been observed to escalate metabolic side effects among psychiatric patients; with olanzapine exhibiting the most significant effects, including weight gain, increased glucose concentrations, and disruption of oxidative homeostasis [3, 4].

The influence of olanzapine on cellular energetics and mitochondrial function is particularly noteworthy [5]. Specifically, olanzapine induces alterations in the mitochondrial network within cells, which may lead to intrinsic mitochondrial dysfunction. Furthermore, it impedes insulin-mediated modifications to the mitochondrial architecture. Importantly, olanzapine promotes mitochondrial network fragmentation while simultaneously inhibiting Protein kinase B (Akt) phosphorylation and insulin-mediated mitochondrial fusion. In addition, the mitochondrial metabolic dysfunction, characterized by a diminished capacity for ATP synthesis, is intrinsically linked to molecular dysfunctions induced by olanzapine [5].

Zinc, identified as a vital trace element, ranks as the second most abundant essential trace mineral present within the human organism [6]. It occupies structural and catalytic roles across all human tissues, particularly regulating DNA synthesis and the activity of antioxidant enzymes. Variations in trace elements, such as zinc and copper, have been correlated with oxidative stress and metabolic dysfunctions [7].

The metabolism of zinc may play a pivotal role in the maintenance of metabolic homeostasis concerning blood glucose levels, as it potentially regulates insulin secretion through the influx of zinc via its transporter ZnT. In accordance with findings by Nicolson et al. (2009), disruptions in zinc signalling may contribute to the pathogenesis of chronic hyperglycemia, which can lead to diabetes [8]. Encouragingly, zinc supplementation has been shown to mitigate and avert hepatocellular apoptosis in individuals exhibiting impaired glucose and lipid metabolism, as well as in those with compensated cirrhosis or chronic hepatitis C [9, 10]. Significantly, zinc enhances mitochondrial respiratory function and attenuates the generation of mitochondrial reactive oxygen species during periods of cellular stress [11].

Interventions utilizing pharmaceutical agents like zinc may prove beneficial in halting the progression of oxidative and metabolic disturbances, as well as liver injury [12], especially in the case of olanzapine, given that perturbations in glucose and lipid metabolism may elicit severe systemic side effects, including hepatic steatosis and fibrosis.

Consequently, the objective of the present study is the evaluation the effects of zinc supplementation on oxidative, inflammatory and metabolic markers including insulin resistance in rats subjected to olanzapine treatment. 

## Materials and methods

### Materials

Merck provided the zinc sulphate. Olanzapine (Integrol^®^) was purchased from Global Napi Pharmaceuticals, Egypt.

### Animals

Twenty-four mature Western Albino rats, weighing 150–170 g at 12 weeks of age, were obtained from National Research Centre Animal House, and have been kept at a constant room temperature of 25°C with a 12-hour light/dark cycle and unrestricted access to food and water. The animals were given a week to acclimate.

### Experimentation design

The experimental design and animal handling during experiments was approved by the animal research ethical committe, National Research Centre of Egypt.

Four groups (*n* = 10) were randomly assigned:

Group 1: As a normal control, rats received no treatment for six weeks.

Group 2: Olanzapine-control group; where rats were given an oral dose of olanzapine (10 mg/kg; p.o, daily) for six weeks [13].

Group 3: Rats were given zinc sulphate (ZnSO_4_; 50 mg/kg, p.o., daily) [14] in addition to olanzapine (10 mg/kg) for six weeks.

Group 4: Rats were given zinc sulphate (ZnSO_4_; 100 mg/kg, p.o., daily) [14] in addition to of olanzapine (10 mg/kg) for six weeks.

Olanzapine and Zinc sulphate were dissolved in saline vehicle.

### Samples

The rats in each group were sedated with ketamine–xylazine at dosage of 100–4 mg/kg IP [15]. At the ending of the treatment period the animals were recoded. This was done for blinding the investigators during sample parameters measurement.

The animals were sacrificed by cervical dislocation as soon as blood was drawn. Using heparinized tubes, blood samples were drawn from a retero-orbitatreal vein, and the separated plasma was kept for biochemical analyses in Eppendorf tubes at -80˚.The liver was detached. Homogenate was prepared by homogenizing a portion of the liver with ice-cooled saline (0.9% NaCl). After that, a cooling centrifuge was used to spin the homogenate for 10 min at 5 °C at 3000 rpm. “Germany, Sigma, Labororzentrifugen”. several analyses were performed using the supernatant [16, 17].

Liver samples were immediately fixed in 10% neutral buffered formalin. paraffin sections (5 μm) were obtained by embedding fixed tissue in paraffin wax, clearing it in xylene, and stained with Hematoxylin and Eosin (H&E) for light microscopy [16, 18].

### Liver oxidative stress parameters

Liver oxidative parameters including MDA, GSH, GSSG, CAT, SOD, AA(Ascorbic acid), coenzyme Q10, Vit E, NO, and Tau(Taurine) were evaluated by the Agilent HP 1200 series (USA) HPLC system consisting of a quaternary pump, a column oven, Rheodine injector with 20 µL loop, and variable-wavelength ultraviolet detector. The resulting chromatogram identified the sample concentration compared to the standard purchased from Sigma Aldrich according to methods by Karatas et al. [19]; Papadoyannis et al. [20]; Yoshida, et al. [21]; Yubero et al. [22].

CAT and SOD activity were measured utilizing the principle of the ability to inhibit target molecule oxidation according to methods in [23], and [24].

### Inflammatory biomarkers

The plasma levels of TNF-α, IL-6 were analyzed using the ELISA method. TNF-α and IL-6 were quantified according to the kit’s protocols R&D Systems, USA. Kit number RTA00 and R6000B respectively.

### Measurement of ATP

Levels of ATP were assessed using the ELISA method in liver tissues, following the kit protocol, Abcam, Cambridge, UK, Kit number ab83355.

### Determination of zinc, iron, and copper in liver tissue

Minerals were determined as described by Imeryuz et al. [25].

### Liver function tests and lipid profile

Blood plasma was used for colorimetric estimation of AST, ALT, ALP GGT, cholesterol, triglycerides, LDL, and HDL by using kits from Salucea Dutch technology in life science – Netherlands.

### Insulin resistance parameters

Blood glucose was considered colorimetrically (Salucea Company kit, Netherlands). Plasma insulin was determined by immunoassay (ELISA), kit number EL0023Ra (Sunlong Biotech Co. Kit, China). Insulin resistance index was computed from the following equation:

$$\eqalign{{\rm{Insulin}} & \,{\rm{resistance}}\,\left({{\rm{HOMA - IR}}} \right) \cr & {\rm{ = }}\,{\rm{fasting}}\,{\rm{glucose}}\,\left({{\rm{mg/dl}}} \right) \cr & \times \,{\rm{fasting}}\,{\rm{insulin}}\,\left({{\rm{mIU/ml}}} \right){\rm{/ 405}} \cr} $$

### Statistical analysis

The results were presented as mean ± standard error (S.E.). The statistical significance was calculated and analyzed by one-way analysis of variance (ANOVA) followed by Tukey’s post hoc test. Pearson correlation coefficient is used to measure of strength of association between different parameters and HOMA-IR. Statistics and graphical presentations were created using GraphPad prism^®^ software (version 6.00 for Windows, San Diego, CA, USA). Values of *p* < 0.05 were considered significant.

## Results

### ZnSO_4_ modulates the hepatic function tests and the lipid profile parameters in rats’ plasma

Olanzapine administration significantly increased liver enzymes and lipid markers compared to the normal control group, with elevations of ALT (+ 174.12%), AST (+ 109.82%), GGT (+ 81.14%), and ALP (+ 46.73%). Lipid profile was also adversely affected with increases in cholesterol (+ 142.61%), LDL (+ 106.76%), and TG (+ 108.63%), while HDL decreased significantly (-55.97%). Treatment with ZnSO_4_ showed dose-dependent improvements, with the 100 mg/kg dose showing superior effects compared to 50 mg/kg. The higher dose reduced ALT (-47.40%), AST (-44.46%), GGT (-38.45%), ALP (-21.43%), cholesterol (-55.70%), LDL (-36.47%), and TG (-46.51%), while significantly increasing HDL (+ 118.55%), bringing these parameters closer to normal control values (Table 1).

**Table 1:** ZnSO_4_ modulates the hepatic function tests and the lipid profile parameters in olanzapine-induced injury in rats

	Liver function tests				Lipid profile			
	AST (U/L)	ALT (U/L)	ALP (U/L)	GGT (IU/L)	Cholesterol (mg/dL)	LDL (mg/dL)	HDL (mg/dL)	TG (mg/dL)
Normal	19.05 ± 0.93	12.83 ± 1.24	81.58 ± 2.8	21.05 ± 1.14	80.17 ± 3.45	50.3 ± 1.55	41.38 ± 2.26	104.33 ± 5.22
Olanzapine	39.97^a^ ± 1.29	35.17^a^ ± 1.84	119.67^a^ ± 2.65	38.13^a^ ± 1.60	194.5^a^ ± 7.76	104^a^ ± 3.19	18.22^a^ ± 0.83	217.63^a^ ± 6.25
Olanzapine + ZnSO_4_ 50	31.62^a, b^±0.83	24.83^a, b^±1.53	102.47^a, b^±2.38	29.02^a, b^±0.97	162^a, b^±5.40	79.43^a, b^±3.41	26.53^a, b^±1.66	153.68^a, b^±6.69
Olanzapine + ZnSO_4_ 100	22.2^b^ ± 0.78	18.5^a, b^±1.40	94.05^a, b^±4.23	23.47^b^ ± 1.09	86.17^b^ ± 3.16	66.07^a, b^±2.20	39.82^b^ ± 1.07	116.35^b^ ± 7.22

Data is presented as the mean ± SE (*n* = 6). Statistical difference is calculated using ANOVA followed by *Tukey’s* test. ^a^ is significantly different from the normal group and ^b^ is significantly different from Olanzapine- control group (*p* ≤ 0.05)

### ZnSO_4_ modulates the hepatic tissue oxidative stress parameters in rats

**Fig. 1:**
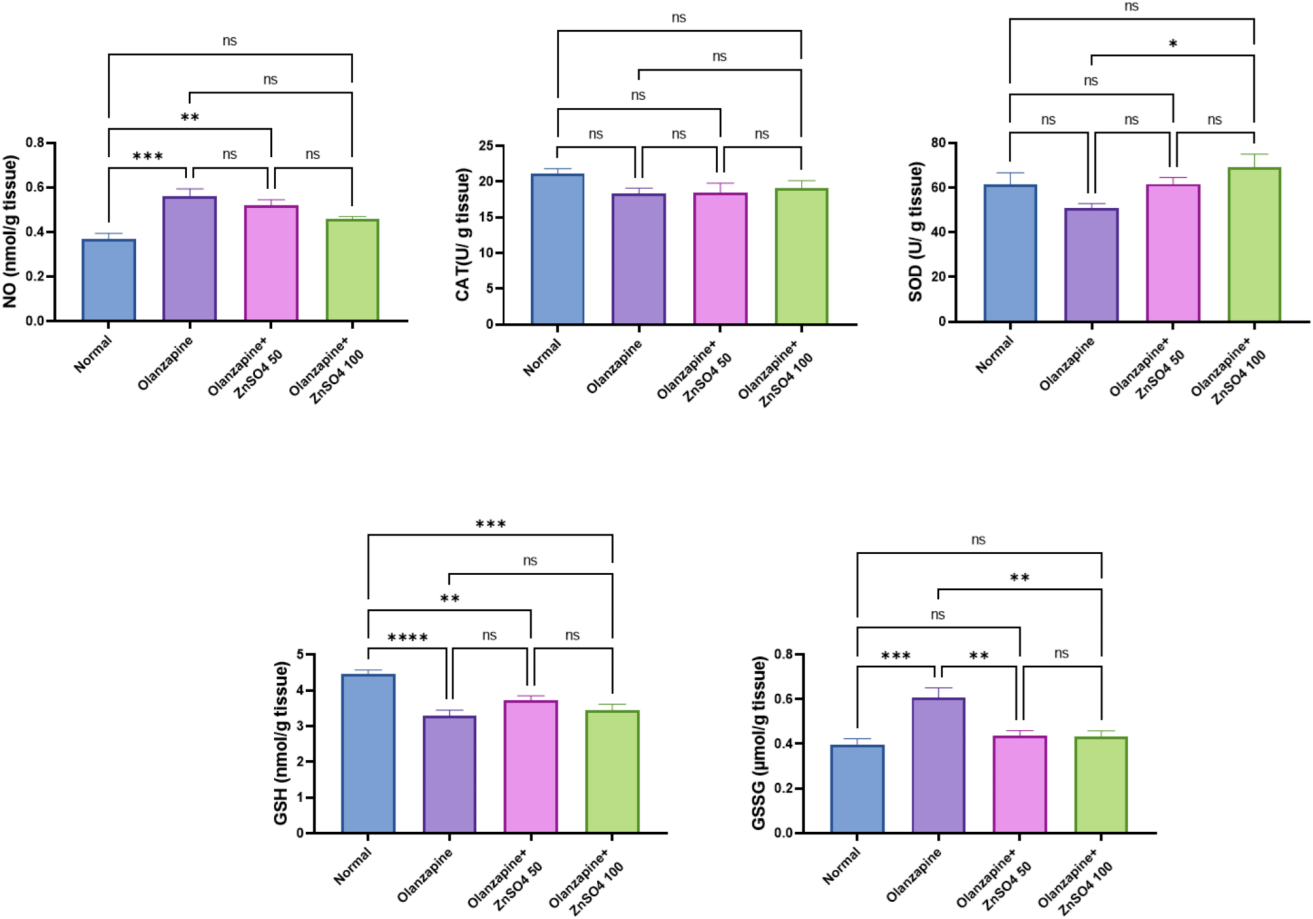
Effects of ZnSO_4_ on the hepatic tissue oxidative stress parameters in olanzapine-induced injury in rats. Data is presented as the mean ± SE (*n* = 6). Statistical difference is calculated using ANOVA followed by *Tukey’s* test (*p* ≤ 0.05)

Olanzapine induced significant oxidative stress, evidenced by decreased antioxidant markers (CAT: -13.05%, SOD: -17.45%, GSH: -26.46%) and increased oxidative stress markers (NO: +51.14%, GSSG: +52.81%). ZnSO_4_ supplementation showed dose-dependent antioxidant effects, with the 100 mg/kg dose demonstrating superior restoration of antioxidant defenses, bringing values closer to normal control levels and showing significant improvement compared to the olanzapine group (Fig. 1).

### ZnSO_4_ reduces plasma and hepatic tissue MDA levels

**Fig. 2:**
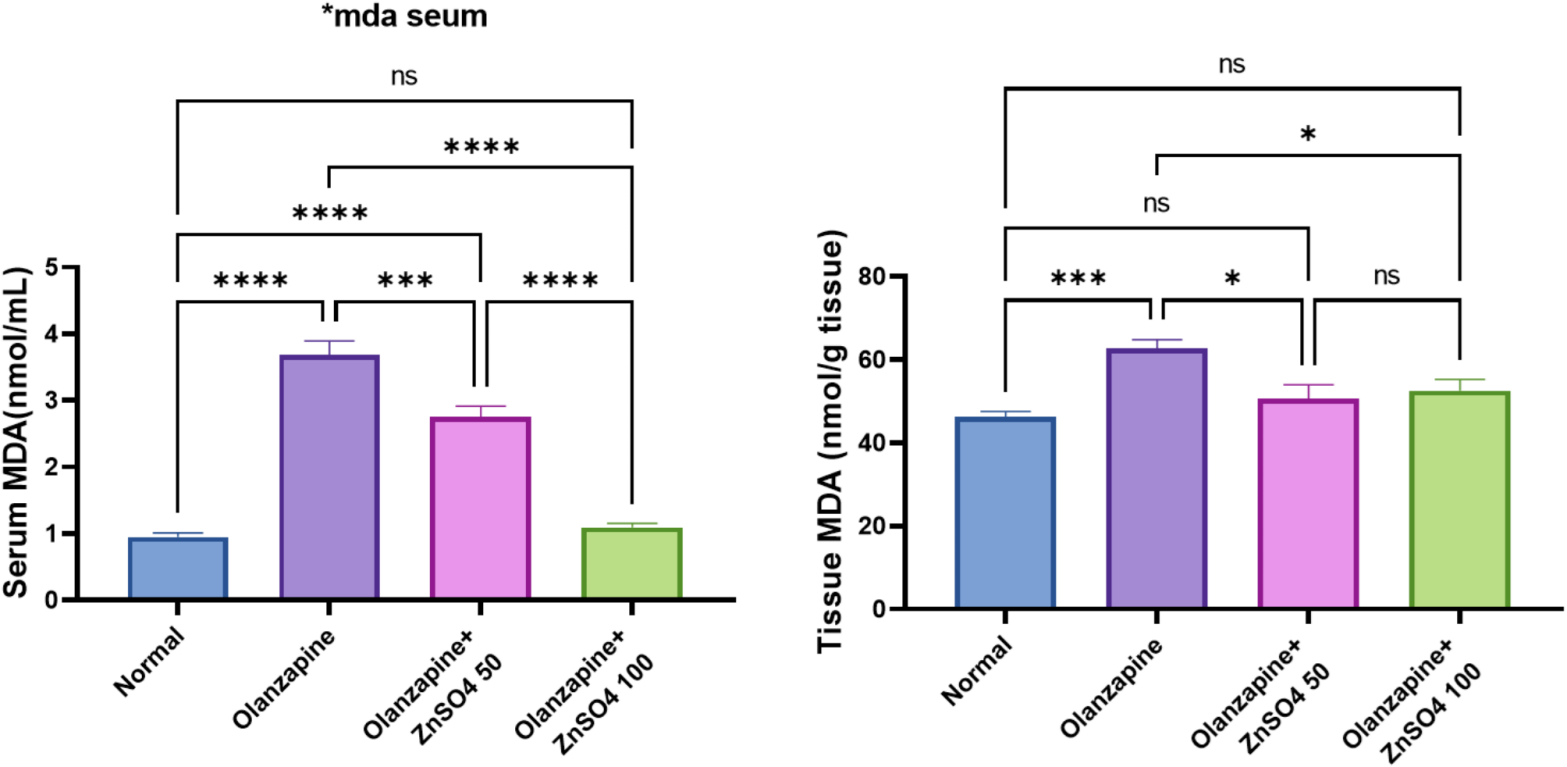
Effects of ZnSO_4_ on the plasma and hepatic tissue MDA levels in olanzapine-induced injury in rats. Data is presented as the mean ± SE (*n* = 6). Statistical difference is calculated using ANOVA followed by Tukey’s test (*p* ≤ 0.05)

Olanzapine significantly increased both plasma and hepatic tissue MDA levels (+ 289.03% and + 35.55% respectively from normal). ZnSO_4_ treatment showed dose-dependent reductions in lipid peroxidation, with the 100 mg/kg dose showing superior effects (-70.35% in plasma and − 16.16% in tissue MDA) compared to the 50 mg/kg dose (-25.33% in plasma and − 19.08% in tissue MDA), bringing these values closer to normal control levels (Fig. 2).

### ZnSO_4_ elevates the hepatic tissue mineral concentrations in rats

**Fig. 3:**
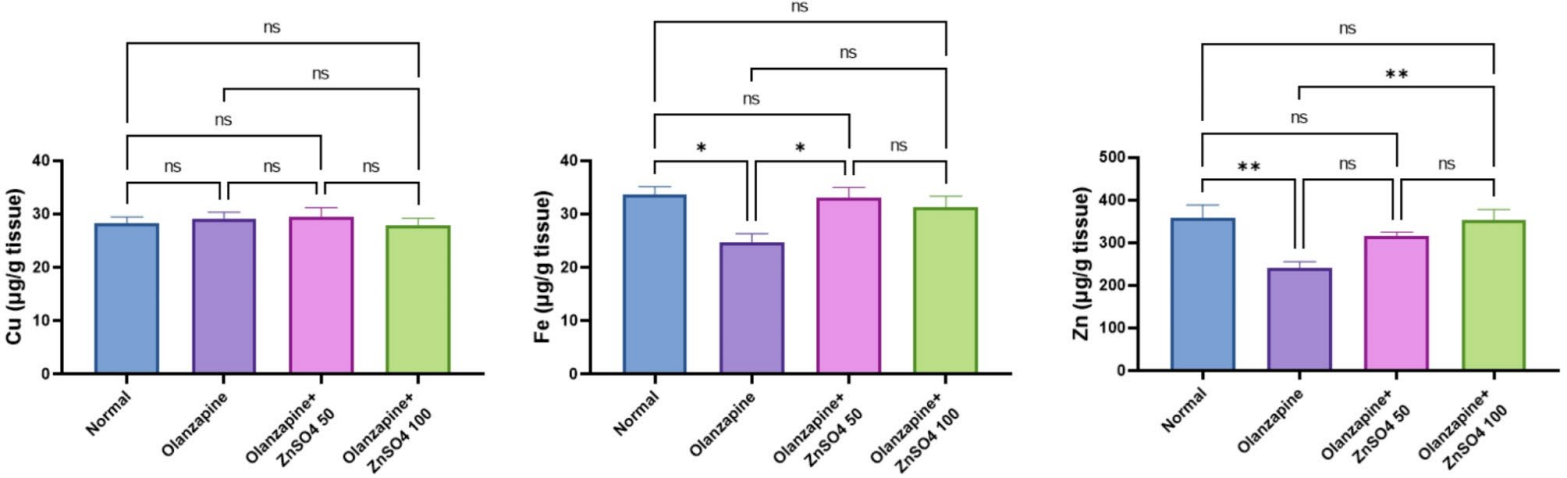
Effects of ZnSO_4_ on the hepatic tissue zinc, iron and copper concentrations in olanzapine-induced injury in rats. Data is presented as the mean ± SE (*n* = 6). Statistical difference is calculated using ANOVA followed by Tukey’s test (*p* ≤ 0.05)

Olanzapine administration significantly decreased zinc (-32.21%) and iron (-26.62%) levels while slightly increasing copper (+ 3.00%) compared to normal controls. ZnSO_4_ supplementation showed dose-dependent restoration of mineral balance, with the 100 mg/kg dose showing superior effects in normalizing zinc (+ 46.07%) and iron (+ 26.49%) levels, while copper didn’t change by the treatments (Fig. 3).

### ZnSO_4_ reduces inflammatory markers in rats’ plasma

**Fig. 4:**
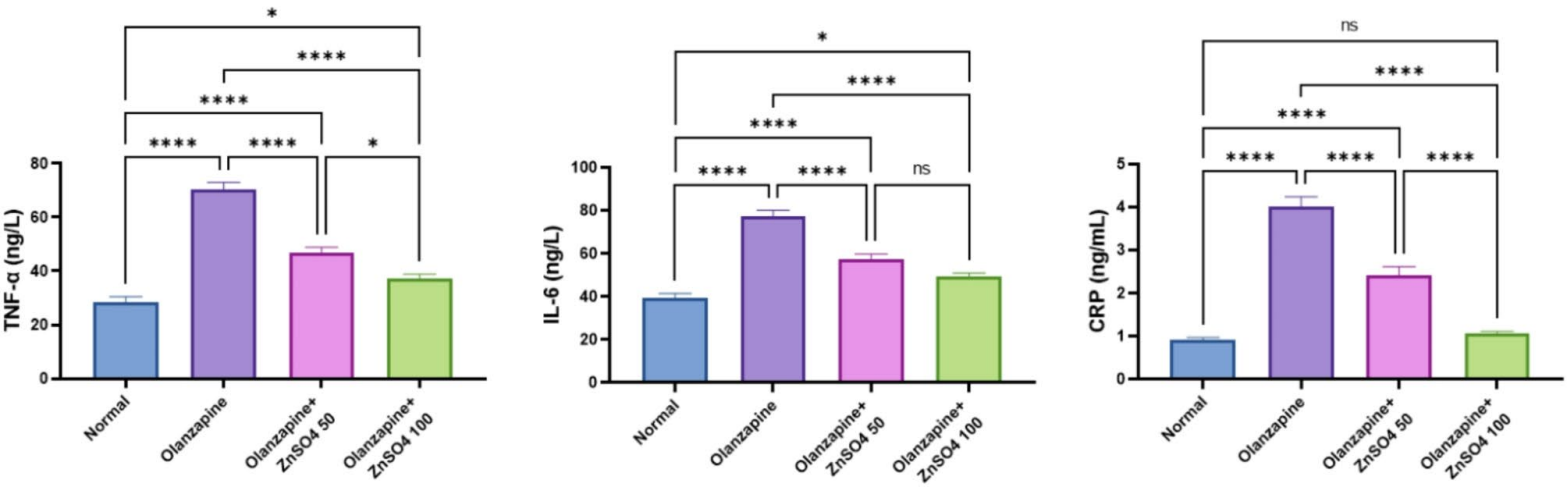
Effects of ZnSO_4_ on the plasma levels of TNF-α, IL-6 and CRP in olanzapine-induced injury in rats. Data is presented as the mean ± SE (*n* = 6). Statistical difference is calculated using ANOVA followed by Tukey’s test (*p* ≤ 0.05)

Olanzapine significantly increased inflammatory markers compared to normal controls (TNF-α: +146.77%, IL-6: +97.42%, CRP: +335.58%). ZnSO_4_ treatment showed dose-dependent anti-inflammatory effects, with the 100 mg/kg dose showing superior reduction in TNF-α (-46.92%), IL-6 (-35.99%), and CRP (-73.55%) compared to the 50 mg/kg dose, making these values reversed to normal control levels (Fig. 4).

### ZnSO_4_ reduces the hepatic tissue energy metabolism in rats

**Fig. 5:**
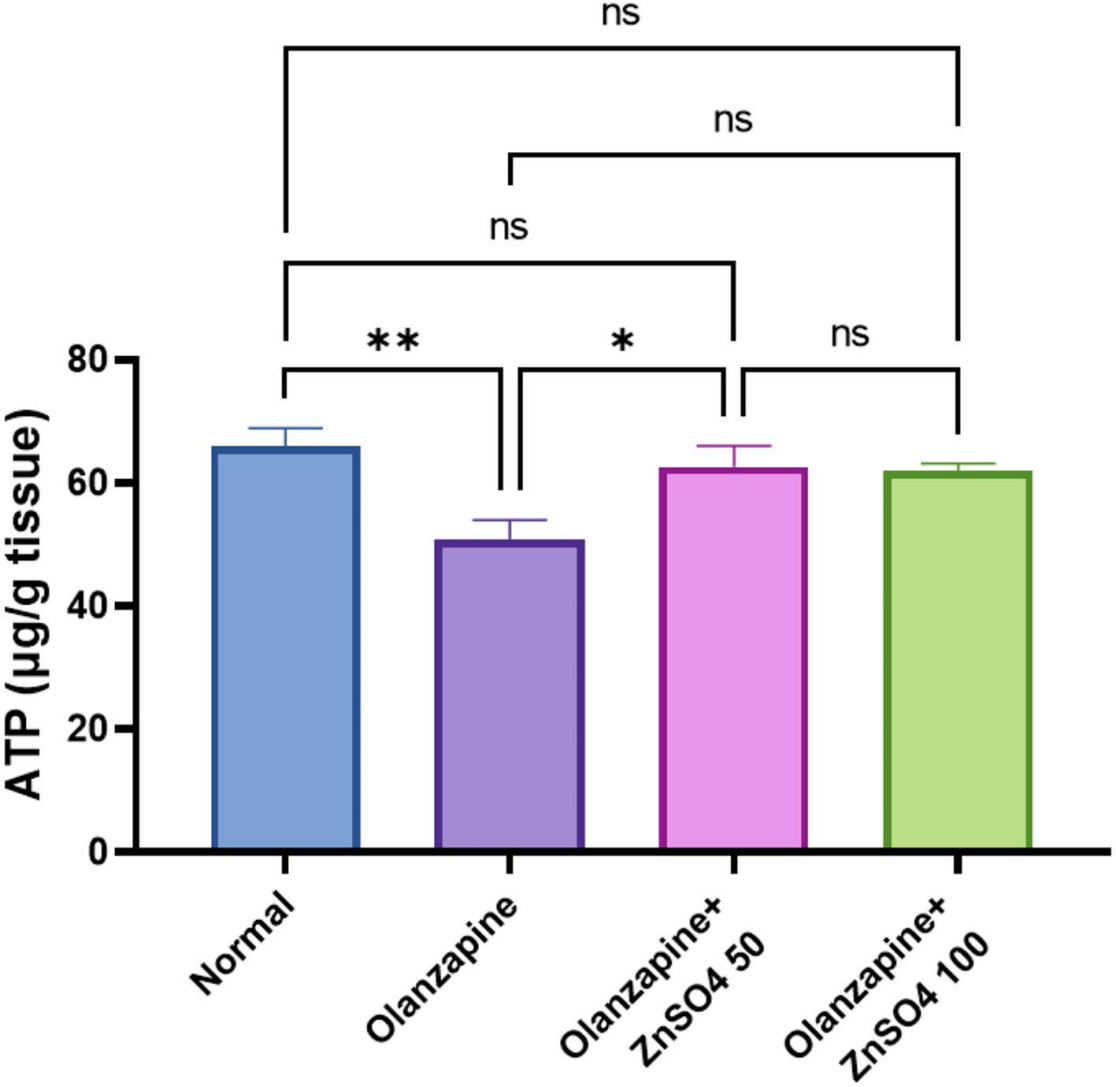
Effects of ZnSO_4_ on the hepatic tissue ATP concentrations in olanzapine-induced injury in rats. Data is presented as the mean ± SE (*n* = 6). Statistical difference is calculated using ANOVA followed by Tukey’s test (*p* ≤ 0.05)

Olanzapine decreased hepatic ATP concentration by -23.13% compared to normal controls. Both doses of ZnSO_4_ showed significant improvement in ATP levels, with the 50 mg/kg and 100 mg/kg doses increasing ATP by + 23.44% and + 21.96% respectively from olanzapine levels, rendering them near normal control values, with no significant difference between the two doses (Fig. 5).

### ZnSO_4_ modulates Ascorbic acid, Vitamin E, coQ10, Taurine and concentrations in hepatic tissues in rats

**Fig. 6:**
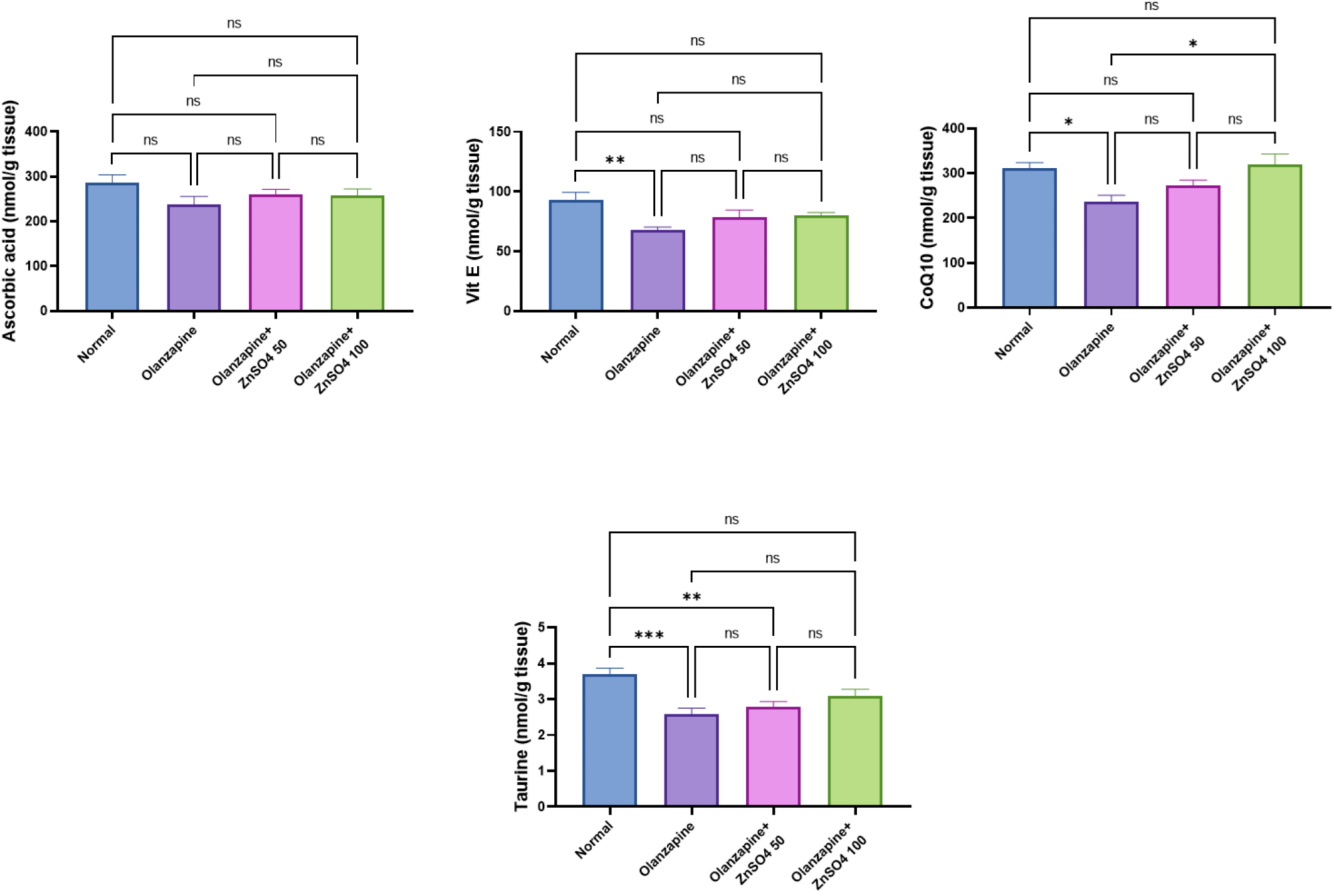
Effects of ZnSO_4_ on the hepatic tissue contents of Ascorbic acid, Vitamin E, coQ10 and Taurine in olanzapine-induced injury in rats. Data is presented as the mean ± SE (*n* = 6). Statistical difference is calculated using ANOVA followed by Tukey’s test (*p* ≤ 0.05)

Olanzapine decreased hepatic concentrations of key antioxidants compared to normal controls (Ascorbic acid: -16.86%, Vitamin E: -26.52%, CoQ10: -23.84%, Taurine: -30.15%). ZnSO_4_ supplementation showed dose-dependent improvements, with the 100 mg/kg dose showing superior restoration of these antioxidants (increases from olanzapine: Ascorbic acid: +7.89%, Vitamin E: +17.17%, +CoQ10: +34.64%, +Taurine: 19.31%), approaching the normal control levels (Fig. 6).

### ZnSO_4_ reduces plasma glucose & insulin levels and modulates insulin resistance in rats

**Fig. 7:**
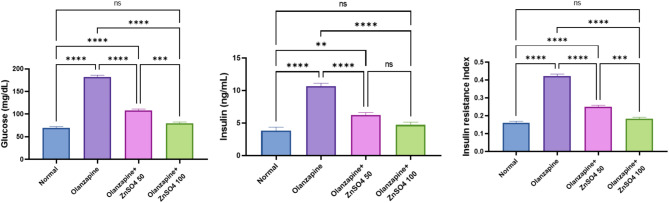
Effects of ZnSO_4_ on the insulin resistance (HOMA-IR) and the plasma levels of glucose & insulin in olanzapine-induced injury in rats. Data is presented as the mean ± SE (*n* = 6). Statistical difference is calculated using ANOVA followed by Tukey’s test (*p* ≤ 0.05)

Olanzapine significantly increased glucose (+ 161.79%), insulin (+ 177.14%), and insulin resistance index (+ 160.85%) compared to normal controls. ZnSO_4_ treatment showed dose-dependent improvements, with the 100 mg/kg dose showing superior effects in reducing glucose (-56.29%), insulin (-55.64%), and insulin resistance (-56.31%) compared to the 50 mg/kg dose, bringing these parameters closer to normal control values (Fig. 7).

### Pearson correlation analyses

**Fig. 8:**
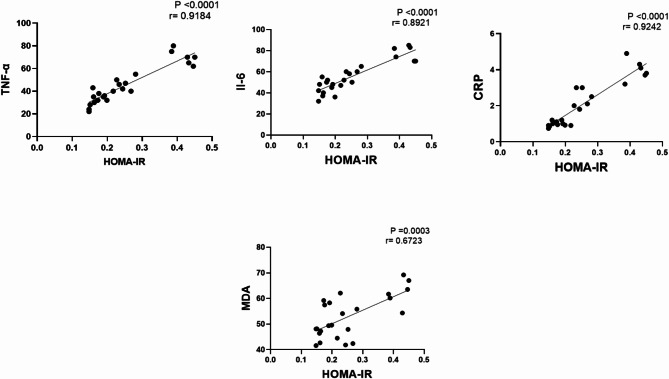
Pearson correlation analysis of different measured parameters against insulin resistance (HOMA-IR) in olanzapine-induced injury in rats

Pearson correlation coefficient was used to measure of strength of group. between different parameters and HOMA-IR values for each group. Data showed that HOMA-IR has a strong positive correlation with TNF-α, IL-6, CRP, MDA. This data suggests a close association between HOMA-IR and the above-mentioned parameters (Fig. 8).

### ZnSO_4_ mitigates histopathological changes in rats’ liver tissues

**Fig. 9:**
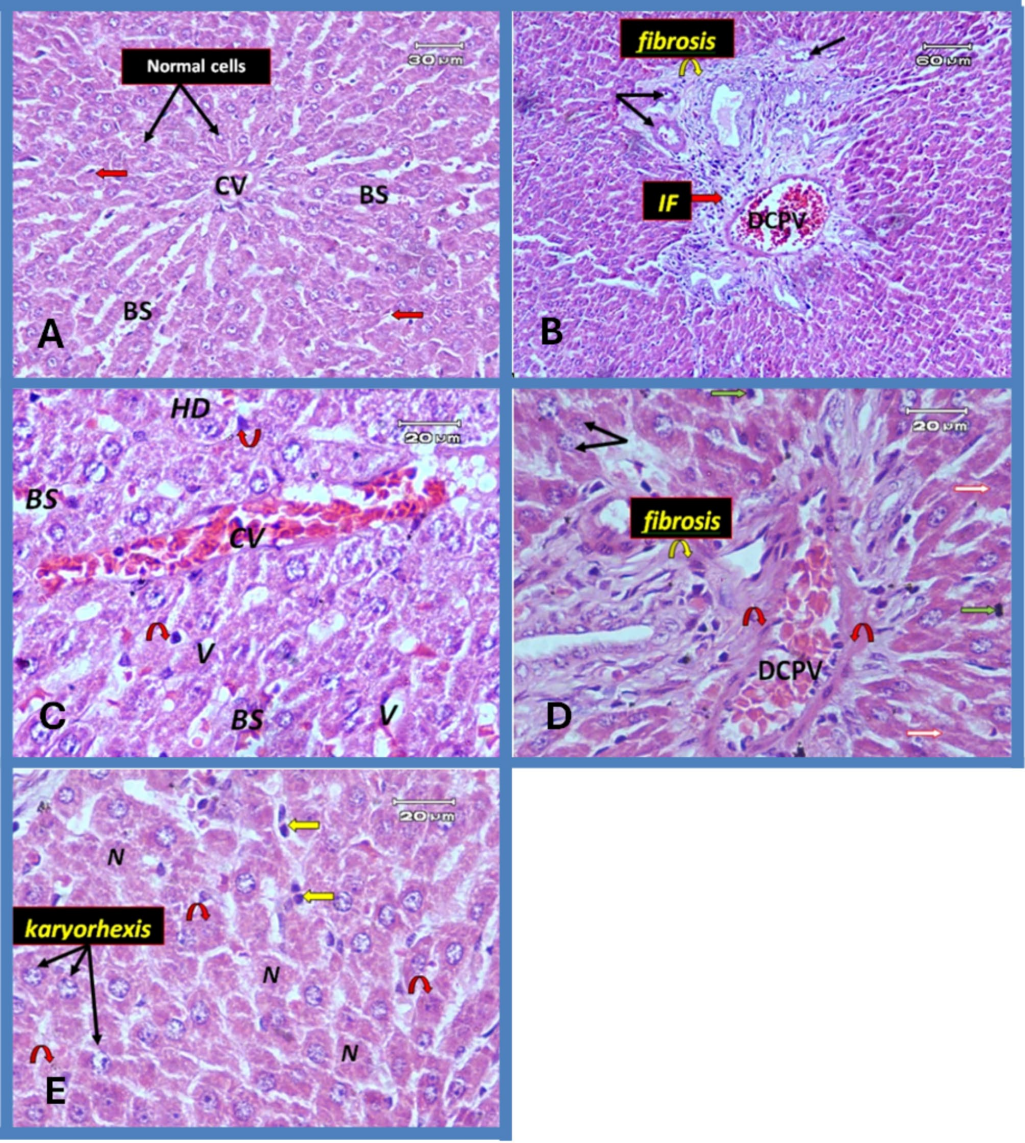
(**A**): A photomicrograph of rat liver of control rats showing normal hepatic architecture. The hepatic lobules containing central vein (CV) and separated by blood sinusoids (BS). The cords of hepatocytes are lined by Kupffer cells (red arrow). (**B**): Photomicrograph of the liver of rats treated with olanzapine only showing dilated and congested portal vein (DCPV), and inflammatory infiltrate around (red arrow), proliferation of bile duct (black arrow), fibrosis (yellow arrow). (**C**): Photomicrograph of the liver of rats treated with olanzapine only (another filed) showing dilated and congested central vein (CV), some hepatocytes with hydropic degeneration (HD), many with vacuolated cytoplasm (V), some apoptotic cells (red arrow), red blood cells in dilated blood sinusoids (BS). (**D**): A photomicrograph of a liver section of rats treated with olanzapine and subjected to zinc at low dose showing the liver still have changes in the form of dilated and congested portal vein (DCPV) with thickening of wall (red arrow) and fibrosis. Singes of degeneration of liver cells such as karyorrhexis (black arrow), karyolysis (white arrow) and apoptosis (green arrow). (**E**): A photomicrograph of a liver section of rat treated with onlazapine and subjected to zinc at high dose showing substantial improvements as compared to group treated with olanzapine only, however, some liver cells appeared degenerated in the form apoptotic cells (yellow arrow), necrosis in foci of hepatocytes (N). Karyorrhexis (black arrow), karyolysis in some nuclei of hepatocytes (red arrow)

Light microscopic examination of tissue sections showed normal architecture of liver. The hepatic lobule consisted of the central vein in the center of the hepatocytes radiating from it towards the periphery lobule. The hepatocyte appeared polyhedral in shape with centrally located rounded vesicular nuclei. Some hepatocytes were binucleated. The cytoplasm of hepatocytes appeared acidophilic with basophilic granules. The blood sinusoids appeared in between hepatocytes and lined by flat endothelial cell. Blood sinusoids also contained Kupffer cells (Fig. 9, A). Histopathological examination of liver sections obtained from rats treated olanzapine only revealed several changes in the form of marked dilation and congested central vein, portal vein with proliferation of bile duct, cellular infiltration around portal vein and fibrosis. In addition, some hypatocytes with cytoplasmic hydropic degeneration and /or ballooning hepatocytes and many of hepatocytes with vacuolated cytoplasm and some apoptotic cells. Dilated blood sinusoids are filled with red blood cells (Fig. 9B, C). Histopathological alteration in liver of rats treated with olanzapine and subjected to zinc at 50 mg/Kg(low dose) revealed the liver tissue still suffer from injures induced by olanzapine in the form of dilated and congested portal with thickening of wall and fibrosis. Singes in degeneration in liver cells such as karyorrheix, karyolyis and apoptosis (Fig. 9D). The histological changes in liver of rats treated with olanzapine and high dose of zinc induced some improved of histological appearances such as no fibrosis, no inflammatory cells, no vacuolation, no hydropic degeneration.The hypatocytes still suffers from deleterious changes induced by olanzapine in the form some liver cells appeared apoptotic and foci of necrosis in some hepatocytes. Karyorrheix and karyolysis in the nuclei of hepatocytes (Fig. 9E).

## Discussion

The current investigation elucidates that zinc supplementation provides substantial protection to hepatic tissues against oxidative stress, inflammatory responses, and metabolic dysregulation induced by olanzapine. The results obtained herein corroborate findings from previous studies involving various tissues [26–29].

As indicators of hepatic cellular damage and the compromise of cellular integrity, our experimental results indicated the efflux of hepatic cellular enzymes, namely alanine transaminase (ALT), aspartate aminotransferase (AST), alkaline phosphatase (ALP), and gamma-glutamyl transferase (GGT), into the systemic circulation. This phenomenon is intricately associated with the generation of reactive oxygen species (ROS), which is acknowledged as a predominant adverse effect of olanzapine [3, 4, 30]. Furthermore, substantial lipid peroxidation products were generated as a consequence of oxidative damage [31].

Our findings demonstrate that olanzapine incites oxidative stress parameters within hepatic tissue, characterized by an elevation in nitric oxide metabolites (NO) and oxidized glutathione disulphide (GSSG), while simultaneously resulting in a reduction of catalase (CAT), superoxide dismutase (SOD) activities, and glutathione (GSH). Remarkably, zinc sulphate exhibited the capability to ameliorate olanzapine-induced oxidative stress, thereby restoring oxidative parameters to baseline levels.

Olanzapine treatment has been associated with elevated malondialdehyde (MDA) concentrations in liver tissues [32].

Moreover, the current study elucidates the rats subjected to olanzapine treatment exhibited a depletion of vitamins C and E, signifying the loss of principal hydrophilic and lipophilic antioxidant capacities; respectively. Importantly, the levels of MDA were significantly attenuated by the administration of zinc sulphate reflecting mitigation of lipid peroxidation. Olanzapine induces oxidative stress and lipid peroxidation via increasing reactive oxygen species that cause cells damage. Further, the observed many pathological injuries in liver of rats given olanzapine can be attributed to increase in oxidative stress [33].

In the same context, the concentration of the amino acid taurine in olanzapine-treated rats demonstrated a marked decline. Taurine serves as a protective agent for cells, functioning as a sulphur-containing amino acid, non-enzymatic antioxidant, and a vital nutrient [34, 35]. According to the findings of this investigation, oxidative cellular damage may adversely influence taurine uptake and processing by hepatic cells [35]. Notably, zinc sulphate effectively reversed the reduction in taurine levels.

Additionally, the antioxidant coenzyme Q10 is crucial for mitochondrial functionality and the maintenance of redox balance [36]. Our examination revealed a decrease in coenzyme Q10 levels within the hepatic tissues of rats administered olanzapine. A similar deficiency of coenzyme Q10 has been reported in individuals with bipolar disorder depression, as noted by Gahangard et al. [37]. Importantly, supplementation with zinc sulphate led to a significant restoration of coenzyme Q10 levels [37, 38]. Collectively, the data strongly support an antioxidant protective role of zinc sulphate against olanzapine-induced liver injury.

Our research elucidates that olanzapine exerts a significant influence on hepatic cellular energetics, as evidenced by the impairment of ATP synthesis. A comparable result was observed in rodents administered with aripiprazole, which similarly exhibited a reduction in ATP production concomitant with hepatic injury [39]. The restoration of ATP levels upon zinc sulphate administration suggests an advantageous outcome for mitochondrial health, given that ATP serves as the principal mitochondrial energy substrate essential for energy dependant biological and metabolic processes within the cell [5, 11].

Indeed, oxidative stress induced by olanzapine in the liver is intricately linked to blood inflammatory markers. Such oxidative stress has the potential to enhance cytokine production either directly or indirectly, with nuclear factor kappa B (NF-κB) playing a critical role in this association, subsequently amplifying the expression of cytokines such as IL-6 and TNFα, thereby exacerbating the inflammatory response [40]. Further, hepatic C reactive protein secretion was augmented by olanzapine, this leads to further NF-κB stimulation and more exacerbation of cytokines secretion [41]. Our findings indicate that zinc sulphate mitigates these inflammatory markers in conjunction with the alleviation of oxidative stress and hepatocellular injury.

Inflammation precipitates a state of functional iron deficiency in various tissues including liver. Whereby iron levels remain stable, yet iron gets sequestered within macrophages. Macrophages recycle iron daily from erythrocytes, either storing it in ferritin or releasing it into circulation via ferroportin. The expression of hepcidin, induced by inflammatory processes, diminishes ferroportin expression, resulting in iron retention within macrophages, decreased absorption of iron, and subsequent hypoferremia. This condition restricts the availability of iron and contributes to the development of anemia [42]. Our findings reveal a reduction in both iron and zinc levels following olanzapine treatment. These observations are closely associated with the persistent inflammation and oxidative cellular stress instigated by olanzapine. Furthermore, zinc sulphate normalized the levels of iron and zinc, indicating both direct and indirect effects on the recovery of these trace minerals.

It is well established that chronic olanzapine medication frequently results in metabolic syndromes, including insulin resistance and impaired glucose tolerance. Olanzapine induces insulin resistance (IR) by suppressing brown adipose tissue thermogenesis, damping expression of mitochondria uncoupling protein-1 and inhibiting glucose transporters, leading to impaired glucose uptake and decreased response to insulin [30]. According to the present study data on blood sugar, insulin resistance index show that zinc sulphate normalizes the perturbation of blood sugar and insulin resistance induced by olanzapine. Our results are in concordance with previous studies that show zinc’s important role in counteracting hyperglycemia and insulin resistance [43] This suggests a beneficial effect of zinc supplementation in hindering olanzapine-induced hyperglycemia. The favourable effect of zinc on olanzapine induced IR can be referred to a decline in inflammatory markers. Reduction or inhibition of inflammation is mostly associated with improvements in insulin resistance and metabolic functions in animal models of obesity and therefore holds promise as a new therapy -linked metabolic disease [44]. Our results indicated a strong positive correlation between IR and both of IL-6, TNF-α,CRP, and MDA of liver. It appears that IR is a possible significant manifestation of the oxidative stress burden and the widespread inflammatory environment [44].

Moreover, the present report confirms that olanzapine administration significantly elevates plasma triglyceride and total cholesterol concentrations while promoting lipid accumulation within the liver. These findings are consistent with the body of evidence regarding the dyslipidemia effects of olanzapine, which arise from disruptions in the regulation of lipolysis and fatty acid oxidation, alterations in the stimulation of 5′AMP-activated protein kinase (AMPK) in both the hypothalamus liver and peripheral tissues, as well as the dysregulation of carbohydrate and lipid utilization as energy substrates [45]. Importantly, zinc sulphate was observed to ameliorate the disturbances in lipid dyslipidemia parameters, suggesting its potential anti-dyslipidemia properties.

## Conclusion

In conclusion, our experimental endeavours were directed towards elucidating the role of zinc sulphate supplementation in minimizing the deleterious effects of the antipsychotic agent olanzapine through promoting the synthesis of both enzymatic and non-enzymatic antioxidants. Furthermore, we sought to rectify the dysregulation of trace mineral homeostasis, attenuate the inflammatory response, and alleviate dyslipidemia, insulin resistance, and hyperglycemia by utilizing zinc co-treatment.

Furthermore, Our research elucidated the significant advantageous function of zinc supplementation in counteracting the concomitant hepatic damage induced by olanzapine in rats.

## Data Availability

No datasets were generated or analysed during the current study.

## Declarations

### Reporting guidelines

The study is reported in accordance with ARRIVE guidelines (https://arriveguidelines.org).
